# Know the Single-Receptor Sensing Limit? Think Again

**DOI:** 10.1007/s10955-015-1412-9

**Published:** 2015-11-23

**Authors:** Gerardo Aquino, Ned S. Wingreen, Robert G. Endres

**Affiliations:** Department of Life Sciences, Centre for Integrative Systems Biology and Bioinformatics, London, United Kingdom; Department of Molecular Biology, Princeton University, Princeton, NJ 08544 USA

**Keywords:** Information processing, Chemosensing, Physical limits, Ligand-receptor binding

## Abstract

How cells reliably infer information about their environment is a fundamentally important question. While sensing and signaling generally start with cell-surface receptors, the degree of accuracy with which a cell can measure external ligand concentration with even the simplest device—a single receptor—is surprisingly hard to pin down. Recent studies provide conflicting results for the fundamental physical limits. Comparison is made difficult as different studies either suggest different readout mechanisms of the ligand-receptor occupancy, or differ on how ligand diffusion is implemented. Here we critically analyse these studies and present a unifying perspective on the limits of sensing, with wide-ranging biological implications.

**Electronic supplementary material:**

The online version of this article (doi:10.1007/s10955-015-1412-9) contains supplementary material, which is available to authorized users.

## Introduction

In 1977, physicists Howard Berg and Edward Purcell published their results on the fundamental biological problem of sensing [1]. The question they addressed was how accurately a biological cell, viewed as a tiny measurement device, can sense its chemical environment using cell-surface receptors. The paper is not only highly cited, but, more importantly, a large fraction of the citations stems from the last ten years, demonstrating how far ahead of its time the study was. In essence, the message of the paper was simple: sensing in the microscopic world boils down to counting molecules, which arrive at the cell surface by diffusion. Humans encounter a similar limit when we try to see in the near dark as our photoreceptors count single photons [2]. Berg and Purcell’s paper has influenced many fields of quantitative biology, including nutrient scavenging [3–5], mating [6], signal transduction [4, 7], gene regulation [8], cell division [9–11], and embryonic development [12]. While there is no disagreement on the importance of knowing the fundamental physical limits of sensing, there has been disagreement on what this limit is, even for a single receptor. The analysis here interprets and unifies these studies to yield a coherent picture of the limits of sensing.

## Overview

**Fig. 1:**
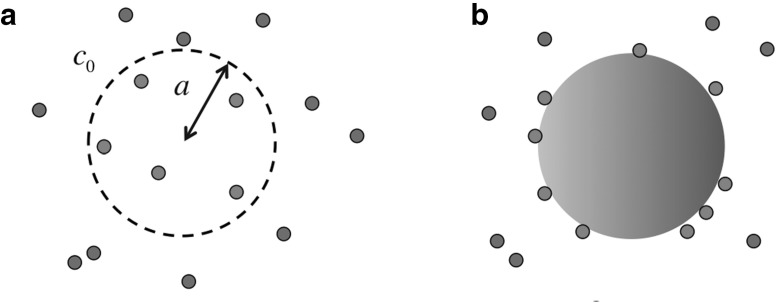
Simple measurement devices for concentration. **a** The perfect monitor is permeable to ligand molecules and estimates the concentration $c_0$ by counting the molecules in its volume during time *T*. **b** The perfect absorber estimates the ligand concentration from the number of molecules incident on its surface during time *T*

To introduce the topic and to build intuition, we follow Berg and Purcell [1] and begin with simple models for measuring ligand concentration $c_0$. The first is the Perfect Monitor [1]. This model assumes a permeable sphere of radius *a*, capable of counting the number of molecules *N* inside its volume (Fig. 1a). For concreteness, the sphere might represent a bacterial cell. Since the molecules diffuse independently, finding a molecule in one small volume element is independent of finding another one in a different small volume element, and so the number of molecules *N* will be Poisson distributed. Since for the Poisson distribution the variance equals the mean, i.e. $\delta N^2=\bar{N}$ (omitting ensemble-averaging brackets for simplicity of notation), we obtain for a single measurement (“snapshot”)

1 $$\begin{aligned} \frac{\delta c^2}{c_0^2}=\frac{\delta N^2}{\bar{N}^2}=\frac{1}{\bar{N}}= \frac{1}{c_0V}, \end{aligned}$$

where $c_0$ is a fixed, given ligand concentration and *V* is the volume of the monitoring sphere. However, if we assume the Perfect Monitor has some time *T* available to make a measurement, the uncertainty in the estimate of the true ligand concentration can be further reduced. In time *T*, the Perfect Monitor can make approximately $M\sim T/\tau _D$ statistically independent measurements, where $\tau _D\sim a^2/D$ is the diffusive turnover time for the molecules inside the sphere. This leads to the reduced uncertainty

2 $$\begin{aligned} \frac{\delta c^2}{c_0^2}=\frac{1}{M\bar{N}}= \frac{1}{(T/\tau _D)c_0V}\sim \frac{1}{Dac_0T}, \end{aligned}$$

where we neglect prefactors for this heuristic derivation. (The exact result is $3/(5\pi Dac_0T)$, which can be derived by considering autocorrelations of the molecules inside the volume [1].)

However, the Perfect Monitor is not the best one can do. A more accurate estimate can be made if each ligand molecule is only measured *once* rather than being allowed to diffuse in and out of the sphere. Thus, we consider a perfectly absorbing sphere [5], estimating concentration from the number of absorbed ligand molecules $N_T$ in time *T*, and find (Fig. 1b)

3 $$\begin{aligned} \frac{\delta c^2}{c_0^2}=\frac{1}{N_T}=\frac{1}{4\pi Dac_0T}<\frac{3}{5\pi Dac_0T} \end{aligned}$$

This Perfect Absorber is thus more accurate than the Perfect Monitor (and even more so for spatial gradient sensing by almost a factor of 10) [5]. This result contrasts with Berg and Purcell’s original suggestion that rebinding previously measured ligand molecules does not increase the uncertainty in measurement [1]. However, one of their many key insights was that a sphere with many absorbing patches for ligand is nearly as good at sensing as a fully absorbing sphere, making room for multiple receptor types with different ligand specificity without sacrificing much accuracy.

**Fig. 2:**
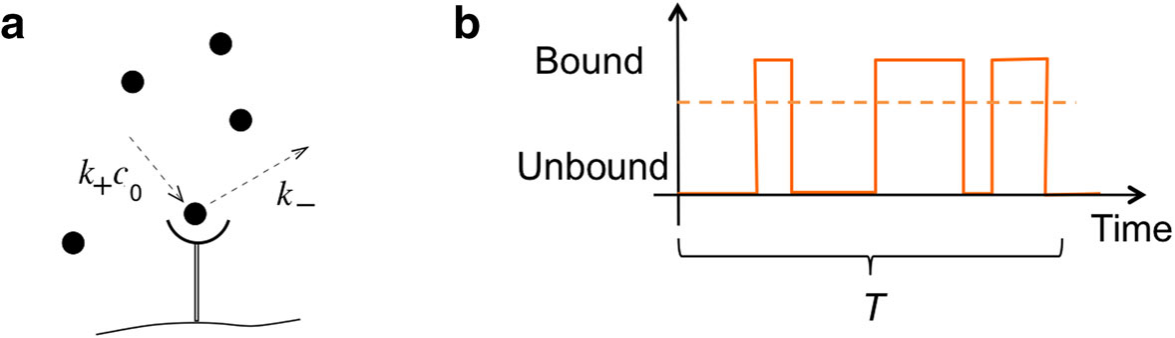
Measuring ligand concentration with a single receptor. **a** A receptor binds ligand with rate $k_+c_0$ when unbound, and unbinds ligand when bound with rate $k_-$. **b** Time series of receptor occupancy during time interval *T*. Berg and Purcell considered the accuracy obtained by taking the average (*dashed horizontal line*)

## Single Receptor Without Ligand Rebinding

The single receptor is the simplest measurement device and thus needs to be thoroughly understood. Unfortunately, different approaches to estimating its sensing accuracy have resulted in significant discrepancies. We first disregard the effects of diffusion and rebinding of previously bound ligands, and just consider ligand binding and unbinding.

Consider the receptor shown in Fig. 2a, which binds ligand with rate $k_+c_0$ when unbound and unbinds ligand with rate $k_-$ when bound. The probability of being bound is then $p=c_0/(c_0 +K_D)$ with $K_D=k_-/k_+$ the ligand dissociation constant. A potential time series of receptor occupancy $\Gamma (t)$ during time *T* is illustrated in Fig. 2b. Berg and Purcell argued that the best a cell can do to estimate the ligand concentration is to average the occupancy $\Gamma (t)$ over time. For such an average, the variance $\delta \Gamma ^2$ was derived from the autocorrelations of occupancy, leading to the relative uncertainty in estimating the ligand concentration

4 $$\begin{aligned} \frac{\delta c^2}{c_0^2}=\left( c_0\frac{\partial p}{\partial c}\right) ^{-2}\delta \Gamma ^2, \end{aligned}$$

where the derivative $\partial p/\partial c$ is the gain or amplification. Naively, one could be tempted to set $\delta \Gamma ^2=p(1-p)$ equal to the variance of a Bernoulli random variable (binomial trials). However, this would correspond to the uncertainty in the concentration estimate following a single instantaneous observation of the state of the receptor, or equivalently to the frequency integral of the noise power spectrum $\delta \Gamma ^2=\int d\omega /(2\pi ) S_\Gamma (\omega )$ (see Supplementary Information for details). This snapshot limit can be improved if we assume $T{>\!\!>}1/(k_+c_0)+1/k_-$, i.e. that the receptor is allowed to average over a time *T* much larger than the correlation time of ligand binding and unbinding. In this case, one can take the low-frequency limit $\delta \Gamma ^2\approx S_\Gamma (\omega =0)/T$ instead, and Eq. 4 leads to the Berg-Purcell limit for a single receptor

5 $$\begin{aligned} \frac{\delta c^2}{c_0^2}=\frac{2\tau _b}{T p}=\frac{2}{\bar{N}} \rightarrow \frac{1}{2Dac_0(1-p)T}, \end{aligned}$$

where $\tau _b=1/k_-$ is the average duration of a bound interval. The simple formulation as $2/\bar{N}$ follows because the average number of binding and unbinding events in time *T* is $\bar{N}=T/(\tau _b+\tau _u)$, where $\tau _u=(k_+c_0)^{-1}$ is the average duration of an unbound interval. The final result in Eq. 5 follows from detailed balance for diffusion-limited binding.

But is it true that averaging receptor occupancy is the best way to estimate concentration? More recently a limit lower than Eq. 5 was found by applying maximum-likelihood estimation to a time series $\Gamma (t)$ of receptor occupancy [13]. Here the probability $P(\Gamma ,c)$ of observing a time series $\Gamma$ is maximised with respect to the concentration *c*. The resulting best estimate of the concentration comes only from the unbound intervals, since only they depend on the rate of binding and thus on the ligand concentration. To obtain a lower limit on the uncertainty the Cramér-Rao bound [14] can be used, leading to

6 $$\begin{aligned} \frac{\delta c^2}{c_0^2}\ge \frac{1}{c_0^2I(c_0)}\rightarrow \frac{1}{N}, \end{aligned}$$

where $I(c_0)=-\partial ^2\ln (P)/\partial c^2$ is the Fisher information evaluated at $c_0$ and averaged over all trajectories with the same *N* (when employing maximum-likelihood estimation it is easier to work with a fixed number of binding/unbinding events *N* than a fixed time *T*). The limit on the right-hand side of Eq. 6 is obtained for long time series for which the inequality becomes an equality. Note, however, that a slightly sharper bound $1/(N-2)$ can be obtained when using a further improved estimator (see Supplementary Information). Eq. 6 shows that the uncertainty in Eq. 5 can be reduced by a factor of two. This is because only unbound intervals carry information about the ligand concentration. In contrast, the bound intervals only increase the uncertainty and hence are discarded by the maximum-likelihood procedure.

What does the maximum-likelihood result imply about tuning receptor parameters to minimise the uncertainty? The minimal uncertainty is obtained for $N_\text {max}$, the maximal number of binding events provided by very fast unbinding ($k_-\rightarrow \infty$). This ideal limit corresponds to the Perfect Absorber from Eq. 3 as every binding event is counted. (However, the increased accuracy comes at the expense of specificity as any ligand molecule dissociates immediately and hence different ligand types cannot be differentiated.) Maximum-likelihood estimation can also be extended to ramp sensing (temporal gradients) [15] and multiple receptors [16, 17].

**Fig. 3:**
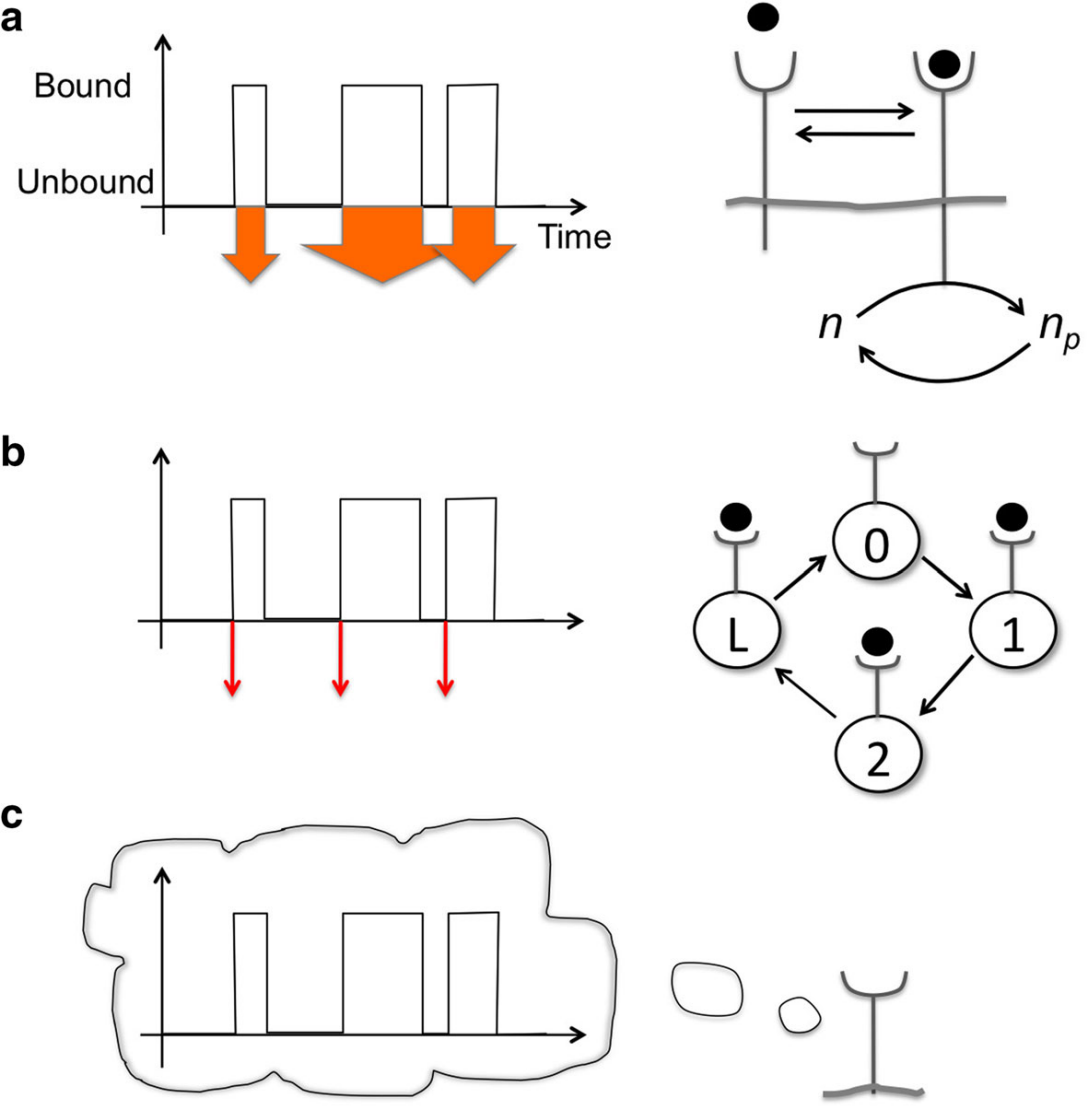
Three schemes of receptor readout. **a** Integrating receptor, which signals while ligand bound (*left*) [1]. For example, the active receptor might phosphorylate a protein with concentration *n*. The concentration of the phosphorylated protein is $n_p$ (*right*). **b** Alternatively, the receptor may signal in generic bursts at onset of ligand binding (*left*) [13]. This scheme can be implemented by an energy-driven cycle of *L* active/bound receptor conformations, which reduces variability (*right*) [18]. **c** A receptor could also retain a memory of previous binding and unbinding events, potentially improving its accuracy of sensing [19]

Adding a downstream signaling molecule cannot increase the accuracy of sensing, in fact this only adds noise. For example, consider an integrating receptor à la Berg and Purcell, which signals while being ligand bound (Fig. 3a) [20]. In this simple network a downstream signaling molecule with concentration *n* is phosphorylated by ligand-bound receptors with the phosphorylated concentration given by $n_p$ with lifetime $\tau$ (beyond this time the protein converts back to the unphosphorylated form). Now, instead of taking the snapshot limit, i.e. the total variance $\delta n_p^2$, we time average to reduce the uncertainty. Specifically, let us assume a long averaging time, that is $T>\!\!>\tau >\!\!>1/(k_+c_0)+1/k_-$, allowing us to use again the low-frequency limit of the corresponding power spectrum. We then obtain (see Supplementary Information for details)

7 $$\begin{aligned} \frac{\delta c^2}{c_0^2}= & {} \left[ {\frac{2}{\bar{N}_\tau }}+ {\frac{2}{\bar{n}(1-p)^2}}\right] {\frac{\tau }{T}}\end{aligned}$$

8 $$\begin{aligned}= & {} {\underbrace{\frac{2}{\bar{N}}}_\text {BP limit}}+ {\underbrace{\left[ \frac{2}{\bar{n}(1-p)^2}\right] }_\text {Poisson-like}} {\underbrace{\frac{\tau }{T}}_\text {time ave}}. \end{aligned}$$

Eq. 8 shows that by integrating receptor output one cannot do better than the Berg-Purcell limit, given by the first term. The second term represents additional Poisson-like noise from number fluctuations of the signaling molecule due to imperfect averaging [21]. For $T{>\!\!>}\tau$ the Berg-Purcell limit is approached from time averaging this noise. While we focus here on averaging in time of stationary stimuli, non-stationary ligand concentrations may be more accurately sensed via non-uniform time averaging, requiring appropriately designed signaling cascades [22].

There has been some confusion about whether sensing actually costs energy. On the one hand, C. H. Bennett pointed out long ago that sensing does not need to cost if done reversibly (and hence extremely slowly) [23]. On the other hand, cells obviously consume energy, e.g. using ATP to phosphorylate proteins. In other words, what energy cost is actually necessary for performing a measurement? As stressed by the authors in [20] the process of sensing in terms of ligand-receptor binding does not need to cost energy if done using an equilibrium receptor in the spirit of Berg and Purcell. However, to accurately infer the external ligand concentration, the cell needs to time average, which cannot be done without consuming energy. This is in line with the Landauer erasure principle [24], which predicts a lower theoretical limit of energy consumption of a computation. In essence, to keep a record of the past for averaging, old information needs to be erased and time-reversal symmetry broken [25]. Time averaging can be implemented by phosphorylation of a downstream protein: when the receptor is bound it phosphorylates and when unbound it dephosphorylates. Since these are energetically driven reactions the reverse reaction, e.g. dephosphorylation by a bound receptor is extremely unlikely, and time averaging is very efficient. The issue of the cost was avoided in Berg and Purcell’s analysis by providing an effective averaging time *T* without specifying how this averaging is achieved.

How is the maximum-likelihood result, Eq. 6, useful? The maximum-likelihood result makes interesting predictions about sophisticated sensing strategies cells might employ. For example, to implement maximum likelihood in the fast unbinding limit a receptor should only signal upon a ligand-binding event as illustrate in Fig. 3b (thin arrows), rather than continuously signaling while ligand is bound (see [26] for further discussion). How can the cell achieve such short and well-defined signaling durations? Reducing variability and achieving determinism requires energy consumption and irreversible cycles [18]. Examples may include ligand-gated ion channels [27] and single-photon responses in rhodopsin of rod cells [28].

Maximum likelihood provides another valuable insight - it shows that information from an estimate and memory from a prior are equivalent, and both can contribute to lowering the uncertainty (Fig. 3c). This kind of receptor “learning” from past estimates can be implemented using the Bayesian Cramér-Rao bound for the uncertainty. Using prior information $I(\lambda )$, one obtains [19]

9 $$\begin{aligned} \frac{\delta c^2}{c_0^2}=-\frac{1/c_0^2}{\underbrace{I(c_0)}_\text {Fisher info.}+ \underbrace{I(\lambda )}_\text {prior}}=\frac{1}{2N}, \end{aligned}$$

assuming the prior had variance 1 / *N*, identical to the actual measurement. Importantly, memory can even help in fluctuating environments if a filtering scheme is implemented by the cell [19]: if the environment fluctuates weakly and/or with long temporal correlations, memory improves precision significantly. If, on the other hand, the environment fluctuates very strongly and/or without any correlations, the cell can still rely on the current measurement (and disregard memory). A form of memory is implemented by receptor methylation in bacterial chemotaxis [29], and in principle memory could be implemented by any slow process in the cell, e.g. the expression of LacY permease in enzyme induction in the lac system [30], or the remodelling of the actin cortex in eukaryotic chemotaxis [31, 32].

## Single Receptor with Ligand Rebinding

So far we have neglected the possibility of rebinding by previously bound ligands. In fact, the role of ligand rebinding in the accuracy of sensing is a tricky issue, because rebinding can introduce non-trivial correlations between binding events. In practice, these correlations can only be included approximately in analytical calculations, and so the question is how to proceed. Originally Berg and Purcell made the reasonable suggestion that a molecule that fails to bind to a receptor may return to the receptor by diffusion and rebind, and that this effect may be included by considering diffusion-limited binding with a renormalised receptor size [1]. However, the question is how to formally separate ligand binding and unbinding from ligand diffusion.

Bialek and Setayeshgar addressed this problem by coupling ligand-receptor binding and unbinding to the diffusion equation [4]. Assuming that the averaging time is long compared to the typical binding and unbinding time, the low-frequency limit can be used. This results in

10 $$\begin{aligned} \frac{\delta c^2}{c_0^2}={\frac{2}{k_+c_0(1-p)T}} +{\frac{1}{\pi Dac_0T}} \end{aligned}$$

with *a* now the size of the receptor. Equation 10 indicates noise contributions from two independent sources. According to Ref. [4], the first term represents binding and unbinding noise and depends on the rate parameters, while the second depends on diffusion and was interpreted as a Berg-Purcell-like noise floor. However, we argue for a different interpretation: For diffusion-limited binding, the first term in Eq. 10 is not zero, but rather $k_+$ needs to be set to a Kramer-like expression, which is proportional to the diffusion constant [33] and an Arrhenius factor at most equal to one [34]. Due to their dependence on the diffusion constant, both terms can be combined [35]. Indeed, for diffusion-limited binding it is the first, not the second term of Eq. 10 that captures the Berg-Purcell limit. Since Berg and Purcell did not consider rebinding by diffusion, the second term constitutes increased noise due to a rebinding correction that does not arise in Berg and Purcell’s derivation [1]. Bialek and Setayeshgar also applied their method to multiple receptors, and showed that the second term can introduce correlations among receptors, as ligand unbinding at one receptor can lead to re-binding at another nearby receptor [35, 36]. Hence, while multiple independent receptors allow for spatial averaging [37], mutual rebinding among different receptors by diffusion increases the uncertainty of sensing.

More recently, Kaizu *et al.* readdressed this problem [38] by applying a formalism developed by Agmon and Szabo for diffusion-influenced reactions [39]. By calculating survival probabilities of bimolecular reactions with a number of simplifying assumptions (see below), they obtained for the relative uncertainty of a single receptor

11 $$\begin{aligned} \frac{\delta c^2}{c_0^2}={\frac{2}{k_+c_0(1-p)T}} +{\frac{1}{2\pi Dac_0(1-p)T}}. \end{aligned}$$

Similar to Bialek and Setayesghar, there are two noise contributions with the first terms in Eq. 10 and 11 formally identical. The second term is, however, different. While the lost factor 2 in the second term in Eq. 11 can be traced to different definitions of the receptor geometry (cubic in Eq. 10 and spherical in Eq. 11), the factor $1-p$ in Eq. 11 is missing from Eq. 10. Due to this factor, both terms of the uncertainty in Eq. 11 diverge if the receptor is fully bound on average ($p=1$), while in Eq. 10 only the first term diverges. Unlike Eq. 10 Kaizu *et al.* made the additional assumption that during a bound interval the external ligand equilibrates. As a result, an unbound ligand molecule cannot diffuse away and rebind at a later time with another ligand bound in between. However, using exact simulations they showed that such delayed rebinding is only a minor effect under biologically relevant conditions. Hence, as the factor $1-p$ in the second term of Eq. 11 also appears in the Berg-and-Purcell limit, Eq. 5, Kaizu *et al.* argue that their result is more accurate than Eq. 10.

However, we propose a slightly different interpretation of Eq. 11. Similar to [4] we suggest that the second term is not the Berg-Purcell limit (Eq. 5) for diffusion-limited binding since the first term captures the Berg-Purcell limit [38]. As described above, for diffusion-limited binding the first term cannot be neglected. This aside, how may the factor $1-p$ in the second term be interpreted? In Kaizu *et al.*’s derivation, diffusion means that a ligand molecule enters the ligand pocket of a receptor without actually binding (hence the factor $1-p$ in the second term since they assume only an unbound receptor can be approached by a ligand molecule). In Bialek and Setayeshgar’s derivation, no such factor appears as the second term describes fluctuations in ligand concentration simply in the vicinity of the receptor. Can further insight into the effects of diffusion be obtained by yet an alternative method?

Maximum-likelihood estimation can also be applied to a receptor with ligand diffusion, albeit only in a special case. The probability of observing a time series of receptor occupancy of *N* binding and unbinding events can be formally written down even with diffusion [13]. However, the rate of binding will depend on the current ligand concentration, which is influenced by the history of all previous binding and unbinding events (even before the first recorded binding event). To estimate the uncertainty, the Cramér-Rao bound can be applied but cannot be evaluated exactly. Nevertheless, for fast diffusion or slow binding an approximate expression can be derived for both 2D and 3D (see Supplementary Information)

12 $$\begin{aligned} \frac{\delta c^2}{c_0^2}\approx \frac{1}{N}\left( 1+2\frac{\Delta c}{c_0}\right) =\frac{1}{k_+c_0(1-p_{1/2})T}+ \left\{ \begin{array}{ll} \frac{\ln (4\pi D/(k_+c_0a^2))}{2\pi Dc_0(1-p_{1/2})T} &{} \text{ for } \text{2D }\\ \frac{1}{\pi Dac_0(1-p_{1/2})T} &{} \text{ for } \text{3D } \end{array}\right. . \end{aligned}$$

In Eq. 12 the average local “excess” ligand concentration $\Delta c$ due to previous binding and unbinding events is $k_+c_0/(4\pi D)\cdot \ln [4\pi D/(k_+c_aa^2)]$ in 2D and $k_+c_0/(2\pi Da)$ in 3D (for the derivation half occupancy $p_{1/2}=1/2$ is required). The ratios in the excess concentration reflect the competition between rebinding and diffusion. As expected, in 2D this concentration decays more slowly to zero with increasing diffusion constant than in 3D, and also the spatial dependence on the receptor size is weaker in 2D than in 3D.

Coming back to the different receptor models with diffusion, the first term of Eq. 12 produces exactly half the uncertainty of the first terms of Bialek and Setayeshgar (Eq. 10) and Kaizu *et al.* (Eq. 11) by utilizing only the unbound time intervals. However, due to factor $1-p$ the second term of Eq. 12 resembles the second term of Kaizu *et al.* (both Eq. 11 and Eq. 12 for 3D use a spherical receptor). This suggests that Kaizu *et al.* is the correct result for the accuracy of sensing by time averaging, while Eq. 12 is the more accurate result when using maximum-likelihood estimation.

**Fig. 4:**
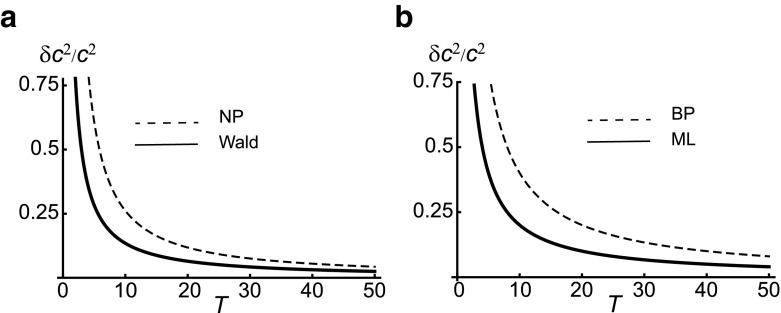
Comparison of decision-making algorithms and fixed-time algorithms. **a** Decision-making algorithms: Wald algorithm (*solid curve*) has lower uncertainty than fixed-time log-likelihood ratio estimation based on the Neyman-Pearson (NP) lemma (*dashed curve*). Uncertainty is calculated by converting decision error into variance. **b** Uncertainty estimates based on direct measurement of ligand concentration in a fixed amount of time: Maximum-likelihood (ML) estimation (*solid curve*) based on the Cramér-Rao bound of Fisher information has only half the uncertainty of the Berg-Purcell (BP) limit (*dashed curve*) for the standard error of the mean concentration. For further details see Supplementary Information

## Single Receptor as a Decision Maker

All the above approaches considered the accuracy based on a fixed measurement time (or number of binding and unbinding events). However, similar to humans, cells might follow a different strategy and approach a problem from a decision-making perspective [40, 41]: either deciding based on existing information or waiting to accumulate more data.

Recently, Siggia and Vergassola considered decision making in the context of cells, proposing that the above maximum-likelihood estimate can further be improved in this way [41]. The simplest implementation of a decision-making strategy is the so-called Wald algorithm [42]. For a single receptor, the Wald algorithm requires calculating the ratio *R* of the likelihoods of the time series of binding and unbinding events of a receptor $\Gamma$ (data), conditioned to either of two hypothesised values of external ligand concentration

13 $$\begin{aligned} R=\frac{P(\Gamma |c_1)}{P(\Gamma |c_2)}. \end{aligned}$$

The cell then concludes that the ligand concentration is $c_1$ if $R\ge H_1$, that the ligand concentration is $c_2$ if $R\le H_2$, or keeps collecting data if $H_1<R<H_2$. $H_1$ and $H_2$ are thresholds that set the probability of error, i.e. concluding the concentration is $c_1$ if the true concentration is $c_2$ and vice versa. This algorithm, by not having a fixed-time constraint, can be shown to be optimal, i.e. the average time to make a decision between the two options is shorter than provided by any other algorithm with the same accuracy (decision-error probability).

How can decision making be compared with maximum-likelihood estimation and the Berg-Purcell limit? Siggia and Vergassola suggested a fixed-time log-likelihood-ratio estimation à la Eq. 13 based on the Neyman-Pearson lemma. Due to the fixed-time constraint the Neyman-Pearson algorithm is in spirit similar to maximum-likelihood estimation. Siggia and Vergassola showed that the Wald algorithm leads to a shorter decision-making time, on average, than the Neyman-Pearson algorithm, and so suggested that the Wald algorithm provides the ultimate limit for sensing.

The result for the Wald algorithm indeed shares properties with the maximum-likelihood estimate and the Berg-Purcell limit. All three reveal a dependence of the measurement (decision) time on the inverse of the square of the difference of concentration (i.e. $\Delta c^2$). Although no decision making is involved in maximum-likelihood estimation or the Berg-Purcell limit, one can still conclude that concentrations $c_1$ and $c_2$ can be distinguished if the measurement uncertainty is smaller than the difference $\delta c^2 < (c_2 -c_1)^2$, and that, assuming either $c_1$ or $c_2$ as the true value, an incorrect decision occurs if measurement returns a value closer to the wrong concentration. This way a decision error can be converted into a type of measurement uncertainty and vice versa (see Supplementary Information for details).

Fig. 4a shows the thus derived uncertainty in measuring a ligand concentration by the Wald algorithm and the Neyman-Pearson lemma as a function of average measurement time. For comparison the maximum-likelihood estimate and the Berg-Purcell limit are shown in Fig. 4b. However, since the fixed-time likelihood algorithms (Neyman-Pearson lemma and maximum-likelihood estimate) do not agree, it is difficult to directly compare Wald with the Berg-Purcell limit. After all, Wald and Neyman-Pearson algorithms are about hypothesis testing and discrimination, while time averaging (Berg-Purcell) and maximum likelihood are about estimation.

What types of algorithm are cells actually implementing? Consider chemotaxis in the bacterium *Escherichia coli* as a prototypical example of chemical sensing. Downstream signaling, especially slow motor switching, could provide a time scale for Berg-Purcell-type time averaging. In contrast, biological systems with hysteresis, that is two different thresholds for activation and deactivation of the downstream pathway, may implement a type of decision-making algorithm. The classical example is the lactose utilisation system in *E. coli*, which can be stimulated by the non-metabolisable ’gratuitous’ inducer TMG [30, 40]. When TMG is high enough enzymes of the lac system become induced. Once induced, however, the TMG level must be reduced below a much lower threshold in order to uninduce the lac system.

## Outlook

While the question of the physical limits of sensing has been around for decades, only over the last few years has the importance of this question become clear and its predictions testable by quantitative experiments [43, 44]. While current work is mostly about chemical sensing, the limits of sensing other stimuli, such as substrate stiffness during durotaxis (or temperature, pH, particles, and combinations of them, etc.) may be next. For such measurements, the role of domain size and spatial dimension are interesting questions. Measurements are often done inside a cell, on 2D surfaces, or along 1D DNA molecules, and correlations due to rebinding depend on these parameters [45].

The question of the limits of sensing has also opened up completely new directions, including the role of active, energy-consuming sensing strategies [18, 20, 46, 47], and hence the importance of nonequilibrium-physical processes in cell biology. This then connects to the Landauer limit of information erasure and cellular computation in general [48, 49]. In this area, important questions are linking information theory, statistical inference, and thermodynamics e.g. in order to produce generalized second laws [50]. Additionally, analysis may move away from only considering receptors to considering receptors and their downstream signaling pathways, and questions of optimal resource allocation in such pathways emerge [25].

Other areas of study have started to benefit from this work as well, such as gene regulation. For instance, why do cells often use bursty frequency modulation of gene expression under stress and in development [51]? This may either reflect a need for accurately sensing and monitoring chemical cues, or simply enhance robustness, e.g. similar to when information is transmitted between neurons by action potentials. The questions whether cells sense at the physical limit and if so, how they reach it, and how to design experiments to answer these questions will occupy us for a while.

## Electronic supplementary material

Supplementary material 1 (pdf 189 KB)
