# Supplementary material for: Know the Single-Receptor Sensing Limit? Think Again
Source: J Stat Phys. 2015 Nov 23;162:1353–64. doi: 10.1007/s10955-015-1412-9 (PMC4761375; doi:10.1007/s10955-015-1412-9)
Supplement: Supplementary file 1 — Supplementary material 1 (pdf 189 KB) [file 10955_2015_1412_MOESM1_ESM.pdf]

Supplementary Information to:

Know the single-receptor sensing limit? Think again.

Gerardo Aquino[1], Ned S. Wingreen[2] and Robert G. Endres[1]

[1]*Department of Life Sciences & Centre for Integrative Systems Biology and Bioinformatics, Imperial College, London,*

[2]*Department of Molecular Biology, Princeton University, Princeton, New Jersey 08544, USA*

Contents

I. Berg-Purcell limit 3

II. Receptor with downstream signalling 4

III. Maximum-likelihood estimation without ligand rebinding 5

IV. Bayesian Cramér-Rao bound including a prior 7

A. Log-normally distributed prior 8

B. Gamma-distributed prior 10

V. Maximum-likelihood estimation with ligand rebinding 11

VI. Uncertainty and decision-making algorithms 15

VII. Neyman-Pearson lemma 16

VIII. Decision-making algorithms vs. Berg-Purcell limit 17

References 18

I. BERG-PURCELL LIMIT

Consider a receptor which binds and unbinds ligand molecules with kinetics for the average occupancy $\Gamma(t)$ given by

$$\frac{d\Gamma}{dt} = k_+ c_0 (1-\Gamma) \; - \; k_- \Gamma, \tag{1}$$

where $k_+ c_0$ is the rate of binding at ligand concentration c and k_- is the rate of unbinding. At steady state, the probability of being occupied is given by $p = c_0/(c_0 + K_D)$ with the ligand dissociation constant $K_D = k_-/k_+$.

What is the uncertainty $\langle \delta c^2 \rangle$ in measuring ligand concentration c_0? If we have the uncertainty in occupancy, $\langle \delta\Gamma^2 \rangle$, we can use error propagation and write for the relative uncertainty in ligand concentration

$$\frac{\langle \delta c^2 \rangle}{c_0^2} = \left(c \frac{\partial p}{\partial c} \right)^{-2} \langle \delta\Gamma^2 \rangle \tag{2}$$

with the term in bracket evaluated at c_0.

To obtain $\langle \delta\Gamma^2 \rangle$ we could be tempted to use the variance of a Bernoulli random variable, given by $p(1-p)$. We would thus obtain $\langle \delta c^2 \rangle / {c_0}^2 = [p(1-p)]^{-1} \geq 4$ and hence at least 400% fractional error. This instantaneous error based on a single measurement in time can formally be derived as follows, which comes in handy later. Equation 1 can be linearised around the steady-state value by introducing $\Gamma(t) = p + \delta\Gamma(t)$ with $\delta\Gamma(t)$ the fluctuations and keeping only terms linear in $\delta\Gamma(t)$. This produces

$$\frac{d(\delta\Gamma)}{dt} = -(k_+ c_0 + k_-)\delta\Gamma + \eta_\Gamma \tag{3}$$

with $\eta_\Gamma(t)$ the fluctuating source, given by white noise with zero average. (That such a linearization is valid for underlying binary dynamics was shown in [1].) Subsequent Fourier transformation from the time to the frequency domain leads to

$$-i\omega\delta\hat{\Gamma} = -(k_+ c_0 + k_-)\delta\hat{\Gamma} + \hat{\eta}_\Gamma, \tag{4}$$

where we applied the Fourier transforms $\Gamma(t) = \int \frac{d\omega}{2\pi} e^{-i\omega t} \delta\hat{\Gamma}(\omega)$ and $\eta_\Gamma(t) = \int \frac{d\omega}{2\pi} e^{-i\omega t} \delta\hat{\eta}_\Gamma(\omega)$. The power spectrum is then obtained as

$$\langle \delta\hat{\Gamma}(\omega)\delta\hat{\Gamma}^*(\omega) \rangle = \langle |\delta\hat{\Gamma}^2(\omega)|^2 \rangle = \frac{Q_\Gamma}{\lambda_\Gamma^2 + \omega^2} \tag{5}$$

with noise strength $Q_\Gamma = \langle |\hat{\eta}_\Gamma(\omega)|^2 \rangle = 2k_+c_0(1-p)$ determined from Poisson statistics and frequency cut-off $\lambda_\Gamma = k_+c_0 + k_-$. The variance is obtained by integrating the power spectrum over all frequencies

$$\langle \delta\Gamma^2 \rangle = \int \frac{d\omega}{2\pi} \langle |\delta\hat{\Gamma}(\omega)|^2 \rangle = \frac{Q_\Gamma}{2\lambda_\Gamma} = p(1-p). \tag{6}$$

In contrast, Berg and Purcell (BP) considered that the receptor has some time T available in order to produce a measurement. We expect the longer the averaging time the more accurate the measurement. Imagine a binary time series of occupancy $\Gamma(t)$ recorded for time T. The BP limit can be derived by estimating the average receptor occupancy p from the time-averaged value $\Gamma_T = 1/T \int dt\, \Gamma(t)$ with the variance given by $\langle \delta\Gamma_T^2 \rangle = \langle \Gamma_T^2 \rangle - \langle \Gamma_T \rangle^2$. The variance can be determined from the autocorrelation function of the occupancy, or equivalently the power spectrum. Specifically, the uncertainty of the occupancy $\delta\Gamma_T^2$ can be calculated by using the low-frequency limit of Eq. 5

$$\langle \delta\Gamma_T^2 \rangle = \frac{\langle |\delta\hat{\Gamma}(\omega \approx 0)|^2 \rangle}{T} = \frac{2p^2(1-p)}{k_+c_0T}. \tag{7}$$

When plugged into Eq. 2, this reproduces the BP limit

$$\frac{\langle \delta c^2 \rangle}{c_0^2} = \frac{2\tau_b}{Tp} = \frac{2}{\bar{N}} \tag{8}$$

with the average number of binding/unbinding events given by $\bar{N} = T/(\tau_b + \tau_u)$ with $\tau_b = k_-^{-1}$ and $\tau_u = (k_+c_0)^{-1}$ the average bound and unbound time intervals.

II. RECEPTOR WITH DOWNSTREAM SIGNALLING

In addition to the receptor, let us consider a signalling molecule that is produced by the ligand-bound receptor, characterised by occupancy $\Gamma(t)$. The kinetics for the copy number $n(t)$ of this signalling molecule is given by

$$\frac{dn}{dt} = k\Gamma \; - \; \tau^{-1}n, \tag{9}$$

where k times Γ is the rate of production and τ is the lifetime of the signalling molecule. At steady state, the copy number is given by $\bar{n} = k\tau p$. Using error propagation once more, we can write

$$\frac{\langle \delta c^2 \rangle}{c_0^2} = \left(c\frac{\partial \bar{n}}{\partial c} \right)^{-2} \langle \delta n^2 \rangle \tag{10}$$

with the term in parentheses evaluated at c_0. The error based on time averaging by T can be derived as before. Equation 9 can be linearised by introducing $n(t) = \bar{n} + \delta n(t)$, producing

$$\frac{d(\delta n)}{dt} = k\delta\Gamma - \tau^{-1}\delta n + \eta_n \tag{11}$$

with $\eta_n(t)$ the fluctuating source, given again by white noise with zero average. Subsequent Fourier transforming from the time to the frequency domain leads to

$$(\tau^{-1} - i\omega)\delta\hat{n} = k\delta\hat{\Gamma} + \hat{\eta}_n, \tag{12}$$

where we applied the additional Fourier transforms $n(t) = \int \frac{d\omega}{2\pi} e^{-i\omega t}\delta\hat{n}(\omega)$ and $\eta_n(t) = \int \frac{d\omega}{2\pi} e^{-i\omega t}\delta\hat{\eta}_n(\omega)$. The power spectrum is then obtained as

$$\langle|\delta\hat{n}^2(\omega)|^2\rangle = \frac{Q_n}{\tau^{-2}+\omega^2} + \frac{Q_\Gamma k^2}{(\tau^{-2}+\omega^2)(\lambda_\Gamma^2+\omega^2)} \tag{13}$$

with noise strength $Q_n = \langle|\hat{\eta}_n(\omega)|^2\rangle = 2kp$ determined from Poisson statistics. The variance is obtained by calculating the time-averaged low-frequency limit

$$\langle\delta n_T^2\rangle = \frac{\langle|\delta\hat{n}(\omega \approx 0)|^2\rangle}{T} = 2\bar{n}\left[1 + \frac{\bar{n}(1-p)}{k_+ c_0 \tau}\right]\frac{\tau}{T}. \tag{14}$$

When plugged into Eq. 10, this produces the following limit

$$\frac{\langle\delta c^2\rangle}{c_0^2} = \left[\frac{2}{\bar{N}_\tau} + \frac{2}{\bar{n}(1-p)^2}\right]\frac{\tau}{T} = \frac{2}{\bar{N}} + \frac{2}{\bar{n}(1-p)^2}\frac{\tau}{T} \tag{15}$$

with $\bar{N}_\tau = \tau/(\tau_b + \tau_u)$ the average number of ligand binding/unbinding events in time interval τ and $\bar{N}$ the average number in time T. The first term in Eq. 15 is the BP limit (cf. Eq. 8). The second term in Eq. 15 is due to Poisson-like number fluctuations in the signalling molecule and could be reduced for large numbers of signalling molecules. Hence the best one can do with an equilibrium receptor is the BP limit.

III. MAXIMUM-LIKELIHOOD ESTIMATION WITHOUT LIGAND REBINDING

Let N be a fixed number of subsequent bound and unbound time intervals (not the average number $\bar{N}$ here), the probability (likelihood) for such a sequence of intervals $\vec{\tau} = (\tau_1, \cdots \tau_N)$ is:

$$P(\vec{\tau}, c) \propto e^{-k_- T_b} e^{-k_+ c T_u} k_-^N (k_+ c)^N, \tag{16}$$

where $T_b = \sum_{i=1}^{N} \tau_b^i$ and $T_u = \sum_{i=1}^{N} \tau_u^i$ are the total bound and unbound times (with $T_b \simeq N\langle\tau_u\rangle$ and $T_u \simeq N\langle\tau_u\rangle$ for N large). Maximising with respect to c leads to:

$$\frac{dP}{dc} = -k_+ T_u P + \frac{N}{c} P = 0 \quad \rightarrow \quad c_{ML} = \frac{N}{k_+ T_u}, \tag{17}$$

i.e. c_{ML} is the concentration value that maximises the likelihood. The uncertainty in concentration measurement can be obtained from the Cramér-Rao bound which connects the uncertainty in concentration to the Fisher information.

Assume a set of measurements $\vec{\tau}$ distributed according to $P(\vec{\tau}, c)$ from which an unbiased estimation of the concentration c_0 is performed. In general it can be shown that given a set of measurements the variance for the expected value of c is bound from below by the Fisher information, i.e.

$$\langle \delta c^2 \rangle = \langle (\hat{c} - c_0)^2 \rangle \geq \frac{1}{I(c_0)}, \tag{18}$$

with $\hat{c}$ the estimated value of the true concentration c_0 and the Fisher information $I(c)$ defined as:

$$I(c) = -\int d\vec{\tau} \frac{\partial^2 \log P(\vec{\tau}, c)}{\partial c^2} P(\vec{\tau}, c) \tag{19}$$

Using Eq. (16) it follows that

$$-\frac{\partial^2 \log P(\vec{\tau}, c)}{\partial c^2} = \frac{N}{c^2}, \tag{20}$$

which, when inserted in Eq. (19), leads to $I(c) = N/c^2$. In the large-N limit the Cramér-Rao bound becomes an equality and translates into the following expression for the uncertainty in concentration sensing at c_0:

$$\frac{\langle \delta c_{ML}^2 \rangle}{c_0^2} = \frac{1}{c_0^2 I(c_0)} = \frac{1}{N}. \tag{21}$$

This result is two-fold lower than the BP limit. The difference is that the maximum-likelihood (ML) estimate considers only the unbound time intervals, as only these contain information about the ligand concentration.

A few comments are in order: The exact expectation value for the estimator can easily be derived from Eq. (17) leading to

$$\langle c_{ML} \rangle = \frac{N}{k_+} \left\langle \frac{1}{T_u} \right\rangle. \tag{22}$$

The probability density for the variable $T_u = \sum_{i=1}^{N} \tau_u^i$, i.e. of having a sequence of N unbound time intervals, is the N-times convolution of the single probability density $k_+ c_0 e^{-k_+ c_0 \tau_u}$,

namely

$$\psi(T_u) = (k_+ c_0)^N e^{-k_+ c_0 T_u} \frac{T_u^{N-1}}{(N-1)!} \tag{23}$$

from which it follows that

$$\left\langle \frac{1}{T_u^m} \right\rangle = \int_0^\infty \frac{1}{T_u^m} \psi(T_u) dT_u = \frac{(N-1-m)!}{(N-1)!}(k_+ c_0)^m. \tag{24}$$

From Eq. (24) one can easily derive $\langle 1/T_u \rangle = \frac{k_+ c_0}{N-1}$, which, by means of Eq. (22), leads to

$$\langle c_{ML} \rangle = c_0 \frac{N}{N-1}. \tag{25}$$

It follows that the estimator is unbiased (i.e. $\langle c_{ML} \rangle = c_0$) only in the asymptotic limit of large N. The results obtained here and in the main text are consistent with this limit. From Eq. (24) evaluated for $m = 2$ one can derive as well the variance for the ML estimator

$$\begin{aligned} \langle \delta c_{ML}^2 \rangle &= \langle c_{ML}^2 - \langle c_{ML} \rangle^2 \rangle = \frac{N^2}{k_+^2} \left\langle \frac{1}{T_u^2} \right\rangle - \langle c_{ML} \rangle^2 = \frac{c_0^2}{N-2} \left(\frac{N}{N-1} \right)^2 \\ &\simeq_{N \gg 1} \frac{c_0^2}{N} + O(1/N^2). \end{aligned} \tag{26}$$

It follows that the exact value for the bound on the variance of the ML estimator is $\frac{c_0^2}{N-2}\left(\frac{N}{N-1}\right)^2$, which coincides with c_0^2/N in the $N \gg 1$ limit apart from terms of order $O(N^{-2})$. The limit of large N is consistent with the assumption that $T_u \gg k_+ c_0, k_-$ implied in the integration carried out in Eq. (24). Note that one can also define the unbiased estimator $c'_{ML} = \frac{N-1}{N} c_{ML}$ for which (using Eq. (26)) one obtains a sharper bound on the variance, given by $\langle (\delta c'_{ML})^2 \rangle = c_0^2/(N-2)$. In summary, by not using the peak value of the likelihood but the mean value, we obtain an unbiased estimator with a slightly lower uncertainty, i.e. $1/(N-2)$ instead of $1/(N-2)[N/(N-1)]^2$.

IV. BAYESIAN CRAMÉR-RAO BOUND INCLUDING A PRIOR

Next we consider cells which preserve a memory of previous environmental conditions. Specifically, the Bayesian Cramér-Rao bound [2–4] allows us to estimate a lower bound to the variance of the expected value of an estimator when a prior distribution for such an estimator is known. Let us call $\hat{c}$ the unbiased estimator of the true concentration c_0. Such a parameter is estimated based on a set of measurements $\vec{\tau}$ distributed according to $P(\vec{\tau}, c)$. If $\lambda(c)$ is the known prior distribution of the parameter c_0, then it can be shown that

$$\langle \delta c^2 \rangle = \langle (\hat{c} - c_0)^2 \rangle \geq \frac{1}{I(\lambda) + I(c_0)}, \tag{27}$$

where averaging on the left-hand side is conducted using the prior distribution, leading to the reduction in uncertainty on the right-hand side. Specifically,

$$I(\lambda) = \int dc\lambda(c) \left[\frac{\partial \log \lambda(c)}{\partial c}\right]^2 \tag{28}$$

is the contribution to the Fisher information from the prior distribution and

$$I(c) = \int dc\lambda(c) \int d\vec{\tau} P(\vec{\tau}, c) \left[\frac{\partial \log P(\vec{\tau}, c)}{\partial c}\right]^2 = -\int dc\lambda(c) \int d\vec{\tau} P(\vec{\tau}, c) \frac{\partial^2 \log P(\vec{\tau}, c)}{\partial^2 c} \tag{29}$$

is the Fisher information about the parameter c_0 given the data $\vec{\tau}$. The second equality in Eq. (29) follows from the relation $\frac{\partial^2 \log P(\vec{\tau},c)}{\partial^2 c} = \frac{P''}{P} - \frac{P'^2}{P^2} = \frac{P''}{P} - \left[\frac{\partial \log P(\vec{\tau},c)}{\partial c}\right]^2$ and that the term $\frac{P''}{P}$ gives zero contribution as can be checked by differentiating with respect to c the normalisation condition

$$\int d\vec{\tau} P(\vec{\tau}, c) = 1. \tag{30}$$

A. Log-normally distributed prior

We first consider the case where the prior distribution has a log-normal form, i.e.:

$$\lambda(c) = \frac{1}{\sigma\sqrt{2\pi}c} \exp\left[-\frac{(\log(c) - \mu)^2}{2\sigma^2}\right] \tag{31}$$

with mean and variance in log-space given by $\langle \log(c) \rangle = \mu$ and $\langle [\log(c) - \mu]^2 \rangle = \sigma$, respectively. In linear space these are given by respective expressions

$$\langle c \rangle = \exp\left(\mu + \sigma^2/2\right) \tag{32a}$$

$$\langle c^2 - \langle c \rangle^2 \rangle = \exp[2(\mu + \sigma^2)] - \exp\left(2\mu + \sigma^2\right) = \exp\left(2\mu + \sigma^2\right)\left[\exp\left(\sigma^2\right) - 1\right]. \tag{32b}$$

Consequently, $I(\lambda)$ is given by:

$$\begin{aligned} I(\lambda) &= \int dc\lambda(c) \left[\frac{\partial \log \lambda(c)}{\partial c}\right]^2 = \int \frac{dc}{c_0^2} \left[\frac{1}{\sigma^2}(\log(c) - \mu) + 1\right]^2 \exp\left[-\frac{(\log(c) - \mu)^2}{2\sigma^2}\right] \\ &= \left(\frac{1}{\sigma^2} + 1\right) \exp\left[-2(\mu - \sigma^2)\right] \end{aligned} \tag{33}$$

Furthermore, $I(c)$ follows from Eq. (19) where $P(\vec{\tau}, c)$, the probability (likelihood) of observing a sequence $\vec{\tau}$ of N bound and unbound time intervals, is given by Eq. (16). Equations (16) and (17) lead to the following expression for $I(c)$:

$$I(c) = \int dc \int d\vec{\tau} P(\vec{\tau}, c) \frac{N}{c^2} \lambda(c). \tag{34}$$

Performing the integration in $\vec{\tau}$ due to the normalisation condition, we obtain:

$$I(c) = \int dc \frac{N}{c^2} \lambda(c) = N \exp[-2(\mu - \sigma^2)]. \tag{35}$$

In conclusion, the uncertainty in ligand concentration with the Bayesian Cramér-Rao bound and a log-normal prior is given by

$$\langle \delta c^2 \rangle \geq \frac{\exp[2(\mu - \sigma^2)]}{N + 1/\sigma^2 + 1}. \tag{36}$$

In the following we deviate slightly from the derivation found in [5]. In this article the prior was assumed to be centred around the true ligand concentration. Here, we assume more conservatively that the prior distribution is centred around the (erroneous) ML value c_{ML} of the concentration obtained from the previous measurement. The variance of the distribution is again given by the standard Cramér-Rao bound from the ML estimation, $\langle \delta c_{ML}^2 \rangle = c_0^2/N$ [6] with c_0 the true value for the concentration. From these assumptions it follows that

$$\langle c \rangle \; = \; \exp\left[\mu + \frac{\sigma^2}{2}\right] = c_{ML} \tag{37a}$$

$$\langle c^2 - \langle c \rangle^2 \rangle \; = \; \big(\exp(\sigma^2) - 1\big) \langle c \rangle^2 = \frac{c_0^2}{N}, \tag{37b}$$

where last equality follows from Eq. (21). We can now express σ^2 in terms of known quantities, noticing that, for large number of events N, from Eq. (37b) it follows:

$$\exp[\sigma^2] - 1 \simeq \frac{1}{N} \quad \rightarrow \quad \sigma^2 \simeq \log\left(1 + \frac{1}{N}\right) \simeq \frac{1}{N}. \tag{38}$$

Inserting this expression for σ^2 into Eq. (36) leads to $\langle \delta c^2 \rangle \geq \frac{c_{ML}^2 e^{-3/N}}{N+1/N+1}$ and therefore to

$$\frac{\langle \delta c^2 \rangle}{c_0^2} \geq \frac{1}{2N}, \tag{39}$$

where c_0 in the denominator is the true value of the concentration, which differs from c_{ML} obtained from previous measurement by at most a correction proportional to $c_0/\sqrt{N}$ (due to Eq. (21)), so that Eq. (39) is correct to leading order for N large, a result identical to the one in [5].

Eq. (39) means that having a prior distribution for N intervals is the same as measuring for $2N$ intervals without a prior distribution. Hence, information is neither lost nor gained. This also means that by using memory (in the form of a prior) a cell can effectively perform longer and hence more accurate measurements without being limited by the actual measurement (averaging) time.

B. Gamma-distributed prior

Alternatively, assume the prior is given by the Gamma distribution

$$\lambda(c) = \frac{c^{\alpha-1}\gamma^{\alpha}e^{-\gamma c}}{\Gamma[\alpha]}, \tag{40}$$

where the parameters α and γ are related to the first and second moment of the distribution:

$$\langle c \rangle = \frac{\alpha}{\gamma} \tag{41a}$$

$$\langle c^2 - \langle c \rangle)^2 \rangle = \frac{\alpha}{\gamma^2}. \tag{41b}$$

For such a prior distribution, $I(\lambda)$ reads:

$$I(\lambda) = \int \left[\frac{\partial \log \lambda(c)}{\partial c}\right]^2 \lambda(c) dc = \int \frac{\lambda'(c)^2}{\lambda(c)} dc = \int_0^\infty dc \frac{e^{-\gamma c}(\gamma c)^{\alpha+1}(\alpha - 1 - \gamma c)^2}{\gamma c^4 \Gamma[\alpha]} = \frac{\gamma^2}{\alpha - 2}. \tag{42}$$

The Fisher information $I(c)$ is determined from Eq. (34) using the Gamma distribution instead of the log-normal distribution

$$I(c) = \int dc \int d\vec{\tau} P(\vec{\tau}, c) \frac{N}{c^2} \lambda(c) = \int \frac{N}{c^2} \lambda(c) dc = N\gamma^2 \int_0^\infty dc \frac{c^{\alpha-3} e^{-\gamma c} \gamma^{\alpha-2}}{\Gamma[\alpha]} = \frac{N\gamma^2}{(\alpha-1)(\alpha-2)}. \tag{43}$$

Consequently, the Bayesian Cramér-Rao bound is given by:

$$\langle \delta c^2 \rangle \geq \frac{1}{\frac{\gamma^2}{\alpha-2} + \frac{N\gamma^2}{(\alpha-1)(\alpha-2)}}. \tag{44}$$

With the same assumptions as done in the previous section, mean value and standard deviation of the prior distribution are set to c_{ML} and c_{ML}^2/N, respectively (see Eqs. (37)), with c_{ML} the ML value for the concentration obtained in previous measurement. Eqs. (41a) and (41b) then imply:

$$\gamma = \frac{N}{c_{ML}} \tag{45a}$$

$$\alpha = N. \tag{45b}$$

In the limit of large N, this leads to relative uncertainty

$$\frac{\langle \delta c^2 \rangle}{c_0^2} \geq \frac{1}{\frac{N^2}{(N-2)} + \frac{N^3}{(N-1)(N-2)}} \simeq \frac{1}{2N}, \tag{46}$$

with again c_0 the true value of the concentration ($c_{ML} \simeq c_0 \pm c_0/\sqrt{N}$). Not surprisingly, this is the same result as obtained in Eq. (39), since both the log-normal and the Gamma distribution can be derived from Gaussian distributed variables. The Gamma distribution is the distribution of the sum of squared normal variables (a.k.a.χ^2 distribution), while the log-normal, as the name suggests, is the distribution of the logarithm of a normally distributed variable.

V. MAXIMUM-LIKELIHOOD ESTIMATION WITH LIGAND REBINDING

Endres and Wingreen [6] applied maximum likelihood (ML) to the problem of estimating the ligand concentration from a time series of ligand-receptor occupancy, but focused on the uncertainty of this measurement without ligand rebinding, i.e. effectively for very fast diffusion. For slower diffusion one should consider possible rebinding of a previously bound ligand molecule, which makes the instantaneous rate of binding a functional of the previous binding and unbinding events. The binding rate can thus be written as $k_+c_0(t, \{t_+, t_-\})$. The rate of unbinding remains k_-, so the ML estimate of concentration still comes entirely from the durations of the unbound intervals.

We quickly review ML estimation of the ligand concentration with ligand rebinding [6]. The probability for a time series to occur given a ligand concentration c_0 is

$$P(\{t_+, t_-\}; c) = \prod_i p_b(t_{+,i}, t_{-,i}) p_-(t_{-,i}) p_u(t_{-,i}, t_{+,i+1}) p_+(t_{+,i+1}), \tag{47}$$

where the probability for a ligand molecule to remain bound from $t_{+,i}$ to $t_{-,i}$ is

$$p_b(t_{+,i}, t_{-,i}) = p_b(t_{-,i} - t_{+,i}) = e^{-k_-(t_{-,i}-t_{+,i})}. \tag{48}$$

The probability for a receptor to remain unbound from $t_{-,i}$ to $t_{+,i+1}$ includes the effect on the binding of the changing concentration of ligand $p_+ \propto k_+(c_0 + \Delta c_i)$ where Δc_i is the perturbation to the ligand concentration from previous binding and unbinding events. Consequently

$$p_u(t_{-,i}, t_{+,i+1}) = e^{-k_+c_0(t_{+,i+1}-t_{-,i}) - k_+ \int_i \Delta c(t')dt'}, \tag{49}$$

where we have expressed the ligand concentration as

$$c(t, \{t_+, t_-\}) = c_0 + \Delta c(t, \{t_+, t_-\}) = c_0 + \Delta c(\{t - t_{-,i}; t - t_{+,i}\}), \tag{50}$$

and used the notation $\int_i dt' = \int_{t_{-,i}}^{t_{+,i+1}} dt'$, $\Delta c(t') = \Delta c(t', \{t_+, t_-\})$, and $\Delta c_i = \Delta c(t_{+,i})$.

The terms can be gathered as before, leading to

$$P(\{t_+, t_-\}; c) \propto e^{-k_- T_b} \cdot e^{-k_+ c_0 T_u} \cdot k_-^N \cdot k_+^N \cdot \prod_i (c_0 + \Delta c_i) e^{-k_+ \int_i \Delta c(t') dt'}. \tag{51}$$

Importantly, all the Δc's depend only on the times of events, not the value of c_0, so $d(\Delta c)/dc = 0$, yielding

$$\frac{dP}{dc} \propto -k_+ T_u P + \sum_i \frac{1}{c_0 + \Delta c_i} P. \tag{52}$$

Setting the above derivative to zero yields an implicit equation for the ML estimate of c_0,

$$\sum_i \frac{1}{c_0 + \Delta c_i} = k_+ T_u, \tag{53}$$

where the sum is over all binding events. Importantly, each Δc_i depends deterministically on all previous binding and unbinding events. For the special case of fast diffusion $D = \infty$ and hence $\Delta c_i = 0$, we obtain [6] $k_+ c_0 = N/T_u = 1/\langle \tau_u \rangle$ where T_u is the total unbound time of the receptor during time T, N is the total number of binding/unbinding events, and $\langle \tau_u \rangle$ is the average unbound time interval.

How accurate is the concentration estimate? Using the Cramér-Rao bound once more, we obtain for the normalised variance

$$\frac{\langle \delta c^2 \rangle}{c_0^2} = -\frac{1}{c_0^2 \left\langle \frac{d^2 \ln(P)}{dc^2} \right\rangle_{c_0}} = \frac{1}{\langle \sum_i (1 + \Delta c_i / c_0)^{-2} \rangle_{c_0}}, \tag{54}$$

where we used P from Eq. 51. Hence, the normalised variance of the ML estimate of the true concentration c_0 is the inverse of the number of unbound intervals with additional corrections in the regime of slow diffusion, due to perturbations in ligand concentration from previous binding and unbinding events.

Equation 54 depends on the average over all trajectories with N binding and unbinding events. Furthermore, each perturbation in ligand concentration, Δc_i, depends on the whole history of binding and unbinding events, making this equation unsolvable. However, we can estimate the effect of diffusion in the limit of slow binding and unbinding, or fast diffusion. Hence, for small $\Delta c_i / c$, we can expand to linear order

$$\frac{\langle \delta c^2 \rangle}{c_0^2} \approx \frac{1}{N} \left(1 + 2 \frac{\langle \Delta c \rangle}{c_0} \right) \tag{55}$$

to simplify the equation for the uncertainty. Equation 55 now contains only a typical perturbation in ligand concentration $\langle \Delta c \rangle$. To estimate this we use the solution of the diffusion equation for a single ligand molecule

$$\Delta_{\pm} c(\vec{r}, t) = \frac{\pm 1}{(4\pi D t)^{d/2}} e^{-\frac{r^2}{4Dt}}, \tag{56}$$

with $+1$ corresponding to an unbound ligand molecule (source) and -1 corresponding to a bound ligand molecule (sink) at $t = 0$. We assume the receptor sits at $\vec{r} = 0$, at which we wish to evaluate perturbation. Here, we provide results for dimensions $d = 2$ and 3.

2-dimensional diffusion: Here we consider $d = 2$ in Eq. 56. To further simplify the calculation of the whole history of binding and unbinding events, we assume all binding events are independent, i.e. only depend on the average rate $k_+ c_0$ and not also on the perturbations. We thus obtain the infinite series

$$\langle \Delta c(t) \rangle = \frac{1}{4\pi D} \left(\left\langle \frac{1}{\tau_1} \right\rangle - \left\langle \frac{1}{\tau_1 + \tau_2} \right\rangle + ...(-1)^{K+1} \left\langle \frac{1}{\tau_1 + \tau_2 + ... + \tau_K} \right\rangle + ... \right), \tag{57}$$

with the most recent (unbinding) event occurring at time $t - \tau_1$ and increasing the overall concentration (source) and the second most recent (binding) event occurring at time $t - (\tau_1 + \tau_2)$ and decreasing the overall concentration (sink), and so on. The averages are performed over the probability of a sequence of K events and then summed in the limit $K \to \infty$ to account for an infinitely long history.

For the special case $p = 1/2$, so that $\langle \tau_u \rangle = \langle \tau_b \rangle = \tau$ and $\lambda = k_+ c_0 = k_-$, each random number τ is generated with the same distribution $\psi(\tau) = \lambda e^{-\lambda \tau}$. In order to evaluate the generic term in the series of Eq. (57) one has first to evaluate the probability density that a given value T_K is obtained for the sum $\sum_{i=1}^{K} \tau_i = T_K$ after K draws of the random variable τ. This probability is given by the K-times convolution of the distribution $\psi(\tau)$, which is:

$$\psi_K(T_K) = \lambda^K e^{-\lambda T_K} \frac{T_K^{K-1}}{(K-1)!}. \tag{58}$$

Then, by using this distribution one can evaluate $\langle \frac{1}{T_K} \rangle$. This allows us to obtain an expression for a generic term with $K \geq 2$ in the sum in Eq. (57), leading to

$$\left\langle \frac{1}{T_K} \right\rangle = \int_0^\infty \frac{1}{T_K} \psi_K(T_K) dT_K = \frac{\lambda}{K-1}. \tag{59}$$

Summing all these contributions for $K \geq 2$ leads to

$$\sum_{K=2}^{\infty}(-1)^{K+1}\frac{\lambda}{K-1} = -\lambda \log 2. \tag{60}$$

The contribution for $K = 1$ has to be calculated separately. In fact, one has to calculate $\langle 1/\tau \rangle = \lambda \int_0^\infty dt\, e^{-\lambda t}/t$, which is the Gamma function $\Gamma(n)$ for diverging parameter $n = -1$. To make progress we realise that the maximal perturbation is the change in concentration due to a single ligand molecule released into a $2D$ area of the order of the size of the binding site, so $\Delta c_{+,max} \simeq 1/a^2 = \frac{1}{4\pi D \tau_a}$. This effectively introduces a minimal time $\tau_a = a^2/(4\pi D)$. As a result, we approximate the integral by

$$\left\langle \frac{1}{\tau} \right\rangle = \lambda \int_{\tau_a}^{\infty} dt \frac{e^{-\lambda t}}{t} = -\lambda \mathrm{Ei}(-x) = -\lambda \left[\gamma + \ln x + \sum_{k=1}^{\infty} (-1)^k \frac{x^k}{k(k!)} \right] \tag{61}$$

with $x = k_+ c_0 \tau_a \ll 1$ and hence $\ln x \ll 0$, and $\gamma \approx 0.57721...$ the Euler-Mascheroni constant. For very small x, γ and the sum can be neglected and the dominant term is the logarithmic part. In this limit it is evident as well that the contribution of the most recent event is much larger than the contribution from all other events in Eq. (60), so that the final result is

$$\langle \Delta c \rangle \approx \frac{k_+ c_0}{4\pi D} \ln \left(\frac{4\pi D}{k_+ c_0 a^2} \right), \tag{62}$$

showing the competition between rebinding with rate $k_+ c_0$ and diffusion to remove the unbound ligand molecule. As expected for 2D, this result only shows a weak dependence on diffusion and receptor size.

3-dimensional diffusion: Here we set $d = 3$ in Eq. 56 and obtain

$$\langle \Delta c \rangle = \frac{1}{(4\pi D)^{3/2}} \left(\langle \tau_1^{-3/2} \rangle - \langle (\tau_1 + \tau_2)^{-3/2} \rangle + ...(-1)^{K+1} \langle (\tau_1 + \tau_2 + ... + \tau_K)^{-3/2} \rangle ... \right). \tag{63}$$

The calculation therefore, under the simplifying assumption $\lambda = k_+ c_0 = k_-$, is analogous to the 2-dimensional case with the only difference that the average $\langle \tau^{-3/2} \rangle = \lambda \int_0^\infty d\tau e^{-\lambda \tau}/\tau^{3/2}$ replaces $\langle \tau^{-1} \rangle$. Also in this case it can be shown that the contribution from the most recent event is dominant as compared to that of all other events (i.e. terms in Eq. (63) with $K \geq 2$). Using the distribution in Eq. (58) it is possible to calculate the contributions from all the terms with $K \geq 2$ in Eq. (63). One obtains for a generic term in the sum:

$$\left\langle \frac{1}{T_K^{3/2}} \right\rangle = \int_0^\infty T_K^{-3/2} \psi_K(T_K) dT_K = \lambda^{3/2} \frac{\Gamma[K - 3/2]}{\Gamma[K]}, \tag{64}$$

which, after summation, leads to:

$$\sum_{K=2}^{\infty}(-1)^{K+1}\left\langle\frac{1}{T_K^{3/2}}\right\rangle = -2(\sqrt{2}-1)\sqrt{\pi}\lambda^{3/2}. \tag{65}$$

The contribution from the most recent event (i.e. first term in Eq. (63)) is given by $\langle\tau^{-3/2}\rangle = \lambda\int_0^\infty d\tau e^{-\lambda\tau}/\tau^{3/2}$. Introducing $x = k_+c_0\tau_a = \lambda\tau_a \ll 1$ as in the 2D case, the evaluation of $\langle\tau^{-3/2}\rangle$ leads to

$$\langle\tau^{-3/2}\rangle = \lambda\int_{\tau_a}^{\infty} d\tau e^{-\lambda\tau}/\tau^{3/2} = 2\lambda^{3/2}\left[\frac{e^{-x}}{\sqrt{x}} - \sqrt{\pi}\text{Erf}\left(\sqrt{x}\right)\right], \tag{66}$$

which, to leading order in $x \ll 1$, leads to

$$\langle\tau^{-3/2}\rangle \simeq \frac{2\lambda}{\sqrt{a^2/(4\pi D)}}. \tag{67}$$

The ratio between the contribution from terms with $K \geq 2$ of Eq. (65) and this contribution amounts to $\sim \sqrt{\lambda\tau_a} = \sqrt{x} \ll 1$, which justifies keeping only the first term in Eq. (63) in the limit $x \ll 1$. The final result is therefore

$$\langle\Delta c\rangle \simeq \frac{1}{(4\pi D)^{3/2}}\langle\tau^{-3/2}\rangle \simeq \frac{1}{(4\pi D)^{3/2}}\frac{2k_+c_0}{\sqrt{a^2/(4\pi D)}} = \frac{k_+c_0}{2\pi Da}, \tag{68}$$

which has a stronger dependence on diffusion and receptor size compared to sensing in 2D. The results for diffusion in 2D and 3D are stated in the main text. Specifically, Eq. 12 in the main text is obtained by using $N = T_u/\langle\tau_u\rangle$ for the number of binding/unbinding intervals with $T_u = (1-p)T$ and $\langle\tau_u\rangle = (k_+c_0)^{-1}$. Note that while we estimate $\langle\Delta c\rangle$ from the whole history of binding events, in our calculation the individual binding events depend only on the average ligand concentration c_0. Hence, similar to other derivations of the uncertainty of sensing by a receptor with ligand rebinding (main text Eqs. (10) [7] and (11) [8]), we calculate the first-order correction to the uncertainty due to ligand diffusion and rebinding. Note also that only the last unbinding event counts for deriving $\langle\Delta c\rangle$ so the exact durations of former unbound intervals do not matter.

VI. UNCERTAINTY AND DECISION-MAKING ALGORITHMS

Rapid and accurate decisions are ubiquitously made in cells motivating modelling of how this timely accuracy is achieved. Here, we follow Siggia and Vergassola to evaluate

the decision time and uncertainty associated with optimal decision-making algorithms [9]. In particular, we want to make a connection between decision-making algorithms and ML estimation/BP limit.

In the case of deciding between two options, e.g. two concentration values c_1 and c_2, it can be shown that the Wald algorithm is, on average, optimal in time. Given a fixed probability that the wrong decision is made, the Wald algorithm makes the decision in the shortest amount of time on average. In this algorithm two fixed thresholds H_1 and $H_2 > H_1$ are given, and at each time step the ratio

$$R = L(\text{data}|c_1)/L(\text{data}|c_2) \tag{69}$$

between the likelihoods conditioned to either option is evaluated. Concentration $c_1(c_2)$ is chosen if $R \leq H_1 (R \geq H_2)$, while data acquisition continues if $H_1 < R < H_2$. The algorithm can be mapped to a diffusion process of the variable $\ln R$ between two absorbing boundaries, corresponding to the thresholds, the values of which are in turn directly connected to the decision-error probability that $c_1(c_2)$ is wrongly chosen when real value is $c_2(c_1)$. In the diffusive approximation for $\ln R$ the average absorption time $\langle T_{\text{abs}} \rangle$, which coincides with the decision time, is given by [9]

$$\langle T_{\text{abs}} \rangle = \frac{x}{V} + \frac{K}{V \sinh(VK/D)} \left[\cosh(KV/D) - e^{-xV/D} \right] \tag{70}$$

with x the initial value, V the drift and D the diffusivity of $\ln R$, and symmetric absorbing boundaries at $x = \pm K = \pm \frac{1}{2} \log(\frac{H_2}{H_1})$.

VII. NEYMAN-PEARSON LEMMA

In the Wald algorithm time is not constrained to be fixed, and this algorithm is optimal in time on average. If time is constrained, i.e. fixed sample or data size, the optimal test is given by the Neyman-Pearson (NP) lemma. When choosing between two options c_1 (reference hypothesis) and c_2 with a criterion A for rejecting c_1 and a given probability of a decision error

$$\alpha = P(A|c_1), \tag{71}$$

i.e. of wrongly choosing c_2 when the data are generated with c_1, the optimal choice is made by rejecting c_1 in favour of c_2 if $R \leq H$ and choosing c_1 otherwise. Again, R is the likelihood

ratio of Eq. (69) and H is an α-dependent threshold (NP lemma). This optimal criterion fulfils by definition the constraint of Eq. (71)

$$\alpha = P(R \leq H|c_1) = \int_{x:R\leq H} L(x|c_1)dx, \tag{72}$$

which shows also that the threshold value H is determined by α. This algorithm is optimal in the sense that any other algorithm based on a different rejection criterion A of the reference hypothesis c_1 but with the same α, will have a smaller probability $P(A|c_2)$ of correctly choosing c_2 (i.e. correctly rejecting c_1) as compared to the analogous NP's probability $P(R \leq H|c_2)$:

$$P(R \leq H|c_2) = \int_{x:R\leq H} L(x|c_2)dx \geq P(A|c_2) = \int_{x:A} L(x|c_2)dx \qquad \forall A \text{ on } x. \tag{73}$$

In other words for all possible data generated with c_2 the NP test will correctly choose c_2 more often than any other test with the same α. For this reason this algorithm may be seen as a maximal-likelihood decision-making algorithm as the likelihood of correctly choosing c_2 is maximal. So while the Wald algorithm is optimal in time on average, for a fixed time the NP algorithm leads to a maximum likelihood of the correct decision.

VIII. DECISION-MAKING ALGORITHMS VS. BERG-PURCELL LIMIT

Berg and Purcell (BP) were the first to derive an estimate for the uncertainty of a measurement of ligand concentration in a fixed time T by a small detecting device, e.g. a cell. Here we consider their model as applied to a single receptor. As discussed, their estimate was later improved by the maximum-likelihood (ML) estimate, showing that the uncertainty is actually smaller by a factor two because only unbound intervals carry information about the external concentration [6]. Estimates for the uncertainty refer to concentration measurements and cannot directly be compared to decision making between two values.

However, one can still assume that in the BP and ML estimates, different concentrations c_1 and c_2 can be told apart if $\sqrt{\langle \delta c^2 \rangle} < |c_2 - c_1|$ and that, assuming either c_1 or c_2 as the true value, a decision error occurs if a measurement returns a value outside one standard deviation from the true value. With these choices for the thresholds one accounts for large fluctuations around the true value, which may lead to wrongly deciding on the other of two possible options for the true value. We are now in a position in which we can attempt to

compare BP and ML estimates with the Wald and NP algorithms. Setting the decision error α of the Wald and NP algorithms equal to the decision error from both the BP and ML estimates introduced above, one obtains minimum value $|c_2 - c_1|$ for fixed value c_1 so that a decision between c_2 and c_1 can be made with error α. This value can in turn be used as a definition of the uncertainty in decision making and compared to the BP and ML estimates.

Specifically, for a given value of T for the Wald algorithm one can use Eq. (70) and, for given c_1, derive the corresponding value of c_2 that can be distinguished in time T with decision error $\alpha = \alpha(K)$, with $x = \pm K$ the absorbing boundaries for the symmetric case. Using this approach, we can plot $(c_2 - c_1)^2$ of the Wald algorithm as a function of T and hence indirectly compare to the uncertainties from BP and ML (Fig. 5). A similar procedure allows us to extract $(c_2 - c_1)^2$ for the fixed-time Neyman-Pearson lemma (which is also shown in Fig. 5).

[1] D. Clausznitzer and R.G. Endres, BMC Syst. Biol. **5**, 151 (2011).

[2] H.L. Van Trees *Detection, Estimation and Modulation Theory* Part I, New York: Wiley (1968).

[3] B.Z. Bobrovsky, E. Mayer-Wolf and M. Zakai, Ann. Stats. **14**: 1421-38 (1987).

[4] R.D. Gill and B.Y. Levit, Bernoulli **1**: 59-79 (1995).

[5] G. Aquino, L. Tweedy, D. Heinrich, R.G. Endres, Sci. Rep. **4**: 5688 (2014).

[6] R.G. Endres and N. Wingreen, Phys. Rev. Lett. **103**, 158101 (2009).

[7] W. Bialek and S. Setayeshgar, Proc. Natl. Acad. Sci. USA **102**, 10040 (2005).

[8] K. Kaizu, W. de Ronde, J. Paijmans, K. Takahashi, F. Tostevin, P.R. ten Wolde, Biophys. J. **106**: 976 (2014).

[9] E.D. Siggia and M. Vergassola, Proc. Natl. Acad. Sci. USA **110**, E3704 (2013).
